# Predictors of persistent moderate and severe food insecurity in a longitudinal survey in Mexico during the COVID-19 pandemic

**DOI:** 10.3389/fpubh.2024.1374815

**Published:** 2024-06-26

**Authors:** Pablo Gaitán-Rossi, Alan Hernández-Solano, Vitervo López-Caballero, René Zurita-Corro, Ximena García-Ruiz, Víctor Pérez-Hernández, Mireya Vilar-Compte

**Affiliations:** ^1^Instituto de Investigaciones para el Desarrollo con Equidad, Universidad Iberoamericana, Mexico City, Mexico; ^2^Tecnológico Nacional de México, Centro Nacional de Investigación y Desarrollo Tecnológico, Cuernavaca, Mexico; ^3^Department of Public Health, Montclair State University, Montclair, NJ, United States

**Keywords:** persistent household food insecurity, machine learning, COVID-19 pandemic, Mexico, longitudinal survey

## Abstract

**Background:**

Household food insecurity (HFI) increased in Latin America by 9% between 2019 and 2020. Scant evidence shows who was unable to recover from the COVID-19 pandemic. Our aim was to use a Machine Learning (ML) approach to identify consistent and influential predictors of persistent moderate or severe HFI over 2 years.

**Methods:**

We use a three-wave longitudinal telephone survey with a probabilistic sample representative of the Mexican population. With a response rate of 51.3 and 60.8% for the second and third waves, the final sample size consisted of 1,074 individuals. The primary outcome was persistent HFI, i.e., respondents who reported moderate or severe HFI in 2021 and 2022. Twelve income-related predictors were measured in 2020, including baseline HFI. We employed 6 supervised ML algorithms to cross-validate findings in models, examined its precision with 4 standard performance indicators to assess precision, and used SHAP values (Shapley Additive exPlanations) to identify influential predictors in each model.

**Results:**

Prevalence of persistent moderate/severe HFI in 2021 and 2022 was 8.8%. Models with only a HFI 2020 baseline measure were used as a reference for comparisons; they had an *accuracy* of 0.79, a *Cohen’s Kappa* of 0.57, a *sensitivity* of 0.68, and a *specificity* of 0.88. When HFI was substituted by the suite of socioeconomic indicators, *accuracy* ranged from 0.70 to 0.84, *Cohen’s Kappa* from 0.40 to 0.67, *sensitivity* from 0.86 to 0.90, and *specificity* from 0.75 to 0.82. The best performing models included baseline HFI and socioeconomic indicators; they had an *accuracy* between 0.81 and 0.92, a *Cohen’s Kappa* between 0.61 and 0.85, a *sensitivity* from 0.74 to 0.95, and a *specificity* from 0.85 to 0.92. Influential and consistent predictors across the algorithms were baseline HFI, socioeconomic status (SES), adoption of financial coping strategies, and receiving government support.

**Discussion:**

Persistent HFI can be a relevant indicator to identify households that are less responsive to food security policies. These households should be prioritized for innovative government support and monitored to assess changes. Forecasting systems of HFI can be improved with longitudinal designs including baseline measures of HFI and socioeconomic predictors.

## 1 Introduction

Household food insecurity (HFI) is defined as the “limited or uncertain availability of nutritionally adequate and safe foods or the limited or uncertain ability to acquire acceptable foods in socially acceptable ways” (1). Copious evidence has shown HFI is associated with worse physical health [i.e., non-communicable diseases as diabetes (2) and hypertension (1)], nutrition outcomes [i.e., obesity, anemia (3), and stunting (4)], higher levels of stress and mental health conditions, such as depression (5), and lower early childhood development outcomes (6). Physical and mental health consequences have even been identified throughout the spectrum of HFI, from mild to severe (7). Moderate or severe food insecurity in the year 2020 affected 30.4% of the world population, but it spiked to 40.9% in Latin America and the Caribbean (8). Population surveys conducted in Latin America between 2019 and 2020 estimated that moderate and severe HFI increased by 9% (8). In Mexico, using monthly telephone surveys, HFI increased by up to 15 percentage points during the early months of the pandemic, rising from 60% in April to 75% in August 2020 (9). The most common factors globally by which the COVID-19 pandemic increased HFI was by declines in income that jeopardized access to food (10)—on average, 36% of the population stopped working during the initial lockdowns, 65% of households reported a decrease in income, and cash transfers were recommended as a key strategy to mitigate HFI (11, 12). Despite the concern and aid toward HFI, most studies were unable to estimate pre-post pandemic persistence in the same households after the pandemic began (13).

Persistent food insecurity is defined as the consistent reporting of HFI in at least two waves of a longitudinal survey (14). Persistent HFI is associated with lower cognitive assessments and a diminished health status (14). Factors exacerbating persistent HFI include being female, being married, and reporting only a “fair” self-assessed health status (15). Persistency of HFI over time can be a relevant indicator to identify those facing conditions that systematically restrain them to be food secure and are likely resistant to common interventions. However, this indicator is rarely monitored or considered when designing and implementing programs to address HFI. The scarcity of longitudinal studies to assess HFI is a key difficulty in estimating the persistence across time in the same households (16, 17). Consequently, more evidence is needed to assess if the predictors of persistent HFI are similar to those of HFI, as regularly measured in cross-sectional surveys.

Machine Learning (ML) techniques have the potential to predict more precise estimates of HFI (18) by enabling the effective modeling of complex relationships (13). These methods have demonstrated superior performance in predicting indicators, such as poverty, compared to traditional models, like linear regressions (19, 20). There is an increasing interest in the food security literature to use ML techniques when high predictive precision is desirable (21, 22). Models combining primary and secondary data suggest that longitudinal data is advisable because previous prevalence of HFI yields a higher explanatory power and lower errors compared with models using only secondary data—up to a 73% accuracy (23). ML models with panel data from Nigeria exemplify the accuracy of these techniques, as it led to a 78–90% accuracy in reporting HFI (24). These new approaches to HFI have some limitations. Data-driven ML techniques tend to have low explanatory power because of the difficulties to identify the importance of single-variables, which hampers its policy value (23). Nevertheless, technical improvements are tackling these shortcomings (25) and longitudinal designs are becoming more common (18), suggesting this is a promising approach to improve the accuracy and usefulness of ML models, while adding to our understanding of HFI.

The aim of the study was to use Machine Learning algorithms to identify constant and influential socioeconomic predictors of persistent moderate or severe HFI in Mexico in 2021 and 2022. It pursued two interrelated objectives: (1) to compare the predictive performance of multiple ML algorithms when a baseline measure of HFI is combined with socioeconomic predictors; and (2) to identify the consistently important variables in predicting persistent HFI in 2021 and 2022.

## 2 Materials and methods

### 2.1 Sample

We used data from the ENCOVID-19 project, whose main objective was to monitor well-being indicators during the COVID-19 pandemic (26). The longitudinal component of the ENCOVID-19 project collected data of the same individuals in the years 2020, 2021, and 2022 through a telephone survey representative of the Mexican population 18 years and older who had a mobile phone—as was a regular research practice during pandemic lockdowns (10). Baseline data was collected between April and August 2020 (*N* = 4,480) during the first lockdown. Follow-up was conducted between July and August 2021 (*N* = 2,300), when the Delta variant was dominant, and the last contact occurred in March 2022 (*N* = 1,400), during the last phase of the Omicron-1 variant. During these two waves no lockdown was enforced (27). Surveys were collected using a one-stage and probabilistic sample, stratified for each state of the country (*n* = 32). Mobile numbers were randomly selected from the most recent version of the National Dialing Plan at the time (28) and data collection was implemented with a Random Digit Dailing technique (29). By 2019, the share of households with access to mobile phones in Mexico was 89%, with high coverage even in rural areas (72.5%) and in households in the lowest income decile (64%) (30). Response rates in the second and third waves of the longitudinal ENCOVID-19 were 51 and 61%, which is standard in these types of designs (31). Due to missing values, the final sample size was 1,074 respondents. An attrition analysis, in Supplementary Table 1, shows there are significant differences between respondents who dropped out the study or had missing values, and those who answered until the third wave and comprise the analytic sample. The group lost in follow-up were younger (3 years), mostly women (+4%), with lower education from the head of household, a lower household’s socioeconomic status, and had higher moderate and severe HFI (+7%).

### 2.2 Variables

HFI was measured using the 8-item adult version of the ELCSA scale (32). It asks if, in the last 3 months, due to a lack of money or other resources, the respondent or any other adult in the household (i) worried to run out of food, (ii) were unable to eat healthy, balanced and nutritious food, (iii) ate only a few kinds of foods, (iv) skipped breakfast, lunch or dinner, (v) ate less than s/he thought should have, (vi) ran out of food, (vii) were hungry but did not eat, and (viii) went without eating for a whole day. Responses to all items are dichotomous (i.e., Yes/No). After computing the total summative score for the eight items, HFI was categorized into two levels: food secure/mild insecurity (total score = 0 to 3), and moderate/severe food insecurity (total score = 4 to 8). We grouped moderate/severe HFI to align our results to Mexico’s multidimensional poverty measure (33). *Persistent* HFI was used as a dependent variable in all models. It is a dummy variable scored as 1 when a respondent reported moderate or severe food insecurity in the 2021 and 2022 waves of the survey. The 2020 variable was used exclusively as a baseline predictor because it has been the most relevant predictor in previous studies (23) and is thus used as a starting point for model comparison. Since mild levels of food insecurity can have a detrimental impact in people’s well-being, as sensitivity analyses in Supplementary Table 2, we repeated our models by categorizing *Persistent* HFI as mild/moderate/severe HFI (total score = 1–8), while food security was scored with a 0 (total score = 0).

Given the sum of evidence showing that declines in income were the main drivers of the increase of HFI during the COVID-19 pandemic (10), the ML approach used 12 socioeconomic variables as predictors (Table 1), all from the 2020 baseline survey using the analytic sample. We dichotomized all variables –except the AMAI index and baseline HFI. Available demographic predictors included age of the head of household (dummy variable—scored as 1 when above the mean of 52 years old) and self-reported sex of the head of household, household size (dummy variable—scored as 1 when above 4 members), indigenous language, or disabilities by any household member, and living in a rural locality. Socioeconomic predictors were the AMAI assets-based socioeconomic status index, where A/B is the highest and E is the lowest (34), and a variable indicating whether the household received government aid. We also included variables related to economic shocks including if the household experienced an income reduction after the COVID-19 quarantine; if someone in the household lost his/her job; and a dummy variable showing if, due to lack of money or resources, the household used coping strategies like evading paying debts, credit cards, or basic household services, borrowing money from family, friends, banks or lenders, pawning objects, or doing some extra activity to get money. The coping strategies variable is not commonly included in population surveys in Mexico, but debt has been found to be a relevant variable during crises (31). Finally, we included the 2020 ELCSA scale in the first round of models categorized into 4 levels (food-secure households, and mild, moderate, and severe HFI).

**Table 1:** Description of predictors from the 2020 baseline survey.

Predictor	Survey question or description	Values	Prevalence (%)
Age	Age of the head of household above the average of 52 years	1 = yes 0 = no	1 = 51.95 0 = 48.04
Sex	Sex of the head of household	1 = woman 0 = man	1 = 31.37 0 = 68.62
Size	Number of household members is above the national average, 4 members	1 = yes 0 = no	1 = 61.26 0 = 38.73
Indigenous	Do you or someone in your household speaks an indigenous language?	1 = yes 0 = no	1 = 12.56 0 = 87.43
Disabilities	Do you or someone in your household have one or more permanent disabilities?	1 = yes 0 = no	1 = 11.82 0 = 88.17
Rural	Do you consider that the location where you currently live is rural or urban?	1 = rural 0 = urban	1 = 28.39 0 = 71.60
Aid	Household receives any type of government aid.	1 = yes 0 = no	1 = 26.81 0 = 73.18
SES	AMAI Household socioeconomic level, where A/B is the highest and E is the lowest.	1 = E 2 = D 3 = D+ 4 = C- 5 = C 6 = C+ 7 = A/B	1 = 2.88 2 = 19.45 3 = 14.24 4 = 12.56 5 = 14.24 6 = 18.71 7 = 17.87
Reduction	Considering the household income from last month, was this income less than it was before the quarantine (February 2020)?	1 = yes 0 = no	1 = 63.22 0 = 36.77
Unemployment	At least one household member lost his/her job in the last month	1 = yes 0 = no	1 = 13.96 0 = 86.03
Coping	Describes whether during the last month due to lack of money or resources, the household took coping strategies such as: stopping paying debts, credit cards, or basic household services, borrowing money from family, friends, banks or lenders, pawning objects or doing some extra activity to get money.	1 = yes 0 = no	1 = 53.53 0 = 46.46
Baseline HFI	Describes the household’s previous food insecurity level	0 = secure 1 = mild 2 = moderate 3 = severe	0 = 36.68 1 = 41.71 2 = 13.96 3 = 7.63

The cutoff point for dichotomizing ‘Size’ was determined using the respective national median of the number of household members, as reported in the 2020 Population and Housing Census conducted by the National Institute of Statistics and Geography (INEGI). The AMAI index is estimated using 5 indicators: (1) education level of head of household; (2) Number of rooms in household; (3) Number of complete bathrooms; (4) Number of employed members aged 14 or over; (5) Ownership of a car or van; (6) Internet connection in household.

### 2.3 Analysis

To estimate and predict persistent moderate/severe HFI in 2021 and 2022 we ran three sets of models with a different combination of predictors: first, only with 2020 baseline HFI; second, we removed HFI and added all the 2020 socioeconomic predictors; and, finally, we used the 2020 baseline HFI and the 2020 socioeconomic predictors together. We start by including baseline HFI because it is the strongest predictor in the literature, so it sets a reference point to compare the added predictive value of the socioeconomic predictors. In the second set of models, we remove baseline HFI to assess a scenario where the only predictors are socioeconomic variables. Finally, the third set reflects a best-case scenario, with all the variables, where we predict persistent HFI with a baseline prevalence and socioeconomic predictors. The hypothesis is that the third set of models yields the highest performance. After describing the percentages of the 2020 predictors in the analytic sample, the analytic strategy followed a series of steps:

1. As is customary in a ML modeling approach, we randomly split the dataset into a training (60%), validation (20%), and testing subsets (20%). The training dataset was used to specify the model parameters, the validation dataset to fine-tune them, and then the testing dataset verified the model performance with new, unseen data (35). An important challenge for the ML approach was the small sample size, specifically the low number of respondents reporting persistent moderate/severe HFI. To address this shortcoming, we used a Synthetic Minority Over-Sampling Technique (SMOTE) where we over sampled the minority class of interest (i.e., the dependent variable) and introduced synthetic examples based on randomly chosen nearest neighbors (36). The inclusion of synthetic cases prompts the ML algorithm to generate larger and less specific decision regions. Consequently, this aids the algorithm in concentrating on information from the minority class, leading to more generalizable results (33).
2. For the estimation we ran 6 supervised models for each of the three sets. Each model used a different ML algorithm tailored to predict binary responses: Logistic Regression (LR), Random Forest (RF), Extreme Gradient Boosting (XGBoost), Support Vector Classifier with a Gaussian kernel function (SVCG), Neural Networks (NN), and Multi-layer perceptron (MLP). We chose these models because they have shown to have high predictive power for dichotomous responses. Moreover, these algorithms are able to handle correlations among variables (23).
  The LR models are the common analytic approach but estimated within the ML framework (i.e., evaluated in the testing dataset). RF and XGBoost are ensemble tree-based models (i.e., supervised, non-parametric classification models), where the algorithms select a predictor, a cut-off point, and then estimates a hierarchy of subsequent predictors that increase the likelihood of identifying the dependent variable (37, 38). The algorithms repeat this process with subsampling and randomly chosen predictors until it can average predictions from all trees. Ensemble tree-based based models have the advantage over other ML algorithms of producing readily interpretable output. Particularly, these models excel when the associations between predictors and the dependent variable is not linear and complex interactions are in play (39). The way these trees manage interactions is by tracing multiple pathways with varied combinations. The XGBoost algorithm uses the errors from previous trees to adjust weights and avoid overfitting.
  The Support Vector algorithms are supervised parametric classification models using deep learning principles (38). Based on input variables, these algorithms create different layers or patterns of variables to predict the dependent variable. Different algorithms used different distributional assumptions (i.e., Gaussian kernel function). Lastly, the NN and MLP algorithms imitate the behavior of interconnected neurons that learn from each other. The algorithms start with a random solution and iterate by optimizing variable weights until the predictions are improved (38). Each network algorithm uses different learning assumptions.
3. We used four post-estimation performance metrics to assess the models: (i) *accuracy,* which is the ratio of the number of correct predictions over total predictions, (ii) *Cohen’s kappa* to reduce the probability of having correct predictions by chance—and is thus preferred over *accuracy* (iii) *sensitivity,* that is a key metric for policy because it shows the probability of identifying a food-insecure household when the household is indeed insecure (the true positive rate), and (iv) *specificity,* which is the probability of detecting a food-secure household when the household is secure (the true negative rate). We compare the metrics between the models first against random estimation (i.e., above 0.5) and then estimating percent change using the first set of models as reference.
4. Finally, we used SHAP values—SHapley Additive exPlanations—to rank the relative contribution of each variable to compare between algorithms. SHAP values are calculated with the weighted sum of the prediction gaps with and without predictors and the weight is estimated by ranking all combinations of predictors (39). To determine the global ranking of the predictors´ importance, we calculate the average of the absolute SHAP values for each variable across all observations in the test dataset, and then we sort them based on their magnitude (23). This approach should be interpreted with caution because these algorithms operate under different assumptions and processes and are therefore not strictly comparable among them. Nonetheless, they illustrate which variables may be consistently relevant across different estimation techniques. The hypothesis is that baseline 2020 HFI will be the most influential predictor of persistent HFI, followed by the index of socioeconomic status, because these variables have been consistently salient in the literature using cross-sectional studies (2, 13, 33).

We used the Python programming language for data preparation and model execution. The models were estimated through the implementation of various Machine Learning frameworks, including TensorFlow, Scikit-learn, and XGBoost. The SHAP values were computed with the SHAP (SHapley Additive exPlanations) library (40).

## 3 Results

In the analytic sample, with responses in the dependent variable from the three waves of the survey (*N* = 1,074), head of households were mostly males (68.6%), with a mean age of 52 years old, and the majority of participants (61.26%) lived in a household with 4 or more residents (Table 1). Indigenous language was spoken by 13 and 12% reported a disability. The sample included respondents from all socioeconomic levels, and 27% reported receiving some type of governmental support. During the first months of the pandemic, in 2020, 63% recognized an income reduction, 14% unemployment in a household member, and 53% of respondents engaged in some financial coping strategy. In 2020, 37% were food-secure households, while 41% reported mild HFI, 14% moderate HFI, and 8% severe HFI. Moderate and severe HFI was 21% in 2021 and 16% in 2022. The prevalence of persistent moderate/severe HFI insecure in 2021 and 2022 was 8.8% (*n* = 96 respondents; 1,315 with the SMOTE synthetic cases).

In Table 2, the first set of models—only with baseline HFI—were slightly better than predicting persistent HFI randomly (i.e., *Cohen’s Kappa* was 0.57). The first set of models were able to correctly identify households with persistent HFI with a *sensitivity* of 0.68 and to correctly identify non-persistent households with a *specificity* of 0.88. The second set of models —only with socioeconomic predictors and without HFI—had a higher precision (i.e., *Cohen’s Kappa* was between 0.40 and 0.67) than the first set of models with a 12 to 17% improvement. Similarly, the *sensitivity* increased to 0.86 and 0.90—an improvement between 26 and 32%—except for Logistic Regression, that decreased to 0.65. The *specificity* decreased in all the models in the second set to 0.75 and 0.82—a reduction in *specificity* between 7 and 14%, when compared with the first set of models. The third set of models—including both, baseline HFI and socioeconomic predictors—were the most precise models for predicting persistent HFI (i.e., *Cohen’s Kappa* was between 0.61 and 0.85), an improvement between 7 and 49% when compared with having HFI-only in the first set of models. The third set of models were also better in *sensitivity*, reaching values from 0.74 to 0.95, an improvement between 8 and 40%. The third set of models marginally improved in *specificity* when compared to the first set of models, with values ranging between 0.85 and 0.92, an increase between 3 and 6%. To sum up, as hypothesized, performance metrics indicate the third set of models, with all variables, are the best performing combination based on *Cohen’s Kappa, sensitivity*, and *specificity*. Moreover, the second set, with socioeconomic predictors only, was strongest in *sensitivity*, while the first set, with baseline HFI only, was strongest in *specificity*.

**Table 2:** Performance metrics for three sets of models using 2020 data to predict persistent household food insecurity in 2021 and 2022.

	Logistic regression	Random forest	XGBoost	SVCG	Neural networks	MLP
**Accuracy**						
1. HFI	0.79	0.79	0.79	0.79	0.79	0.79
2. SES Predictors	0.70	0.83	0.84	0.84	0.82	0.83
3. SES Predictors and HFI	0.81	0.92	0.90	0.92	0.90	0.90
**Cohen’s Kappa**						
1. HFI	0.57	0.57	0.57	0.57	0.57	0.57
2. SES Predictors	0.40	0.67	0.67	0.67	0.64	0.66
3. SES Predictors and HFI	0.61	0.85	0.80	0.84	0.80	0.80
**Sensitivity**						
1. HFI	0.68	0.68	0.68	0.68	0.68	0.68
2. SES Predictors	0.65	0.88	0.86	0.90	0.87	0.90
3. SES Predictors and HFI	0.74	0.93	0.93	0.95	0.91	0.95
**Specificity**						
1. HFI	0.88	0.88	0.88	0.88	0.88	0.88
2. SES Predictors	0.75	0.80	0.82	0.78	0.77	0.77
3. SES Predictors and HFI	0.86	0.92	0.87	0.90	0.89	0.85

HFI, Household Food Insecurity measured with the adult-version of the ELCSA scale; SES, socioeconomic status measured with the assets-based AMAI index; XGBoost, Extreme Gradient Boosting; SVCG, Support Vector Classifier with a Gaussian kernel function; MLP, Multi-layer perceptron.

**Figure 1:**
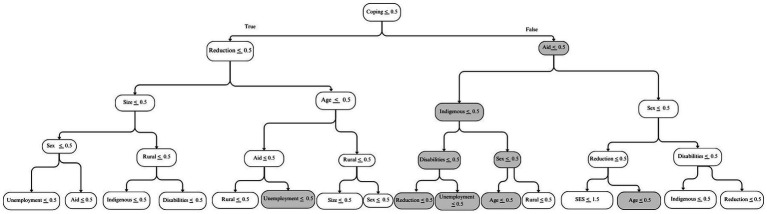
Five-node diagram of the tree with the highest sensitivity value using a Random Forest algorithm in a model without baseline household food insecurity. Nodes in gray indicate concentrations of the sample with higher percentages of persistent HFI. Pathways start with the first node, coping strategies, and show the threshold. When the condition in the threshold is true, the pathway goes to the left; it goes right if the condition is not met.

**Figure 2:**
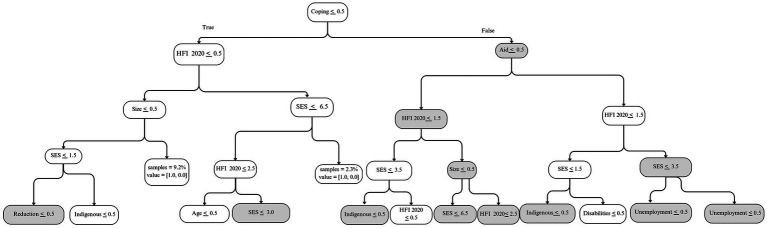
Five-node diagram of the tree with the highest sensitivity value using a Random Forest algorithm in a model with baseline household food insecurity.

While all ML algorithms showed similar results in the performance metrics, the ones with the highest *Cohen’s Kappa* and *sensitivity* were the Random Forest and the Support Vector Classifier with a Gaussian kernel function (SVCG). As an example, Figure 1 shows the tree that maximizes the sensitivity metric in the test subset among the trees generated by the Random Forest algorithm, which were estimated from the second set of models, without baseline HFI. Nodes in gray indicate where the sample has a higher percentage of households with persistent HFI. Each node includes a condition that splits the sample and maximizes the prediction. Pathways are interpreted top-down, where upper nodes are more relevant in predicting the outcome. The pathway goes to the left when the condition is *true* and to the right when the condition is *false*. For example, following the gray pathway, a household engaging in coping strategies, that is not receiving government aid, with an indigenous background, and a male respondent, is at a higher risk of experiencing persistent HFI. Similarly, the tree that maximizes the sensitivity metric in the third set of models shows that a household engaging in coping strategies, not receiving government aid, and reporting moderate/severe HFI in 2020 is at a higher risk of experiencing persistent HFI in 2021 and 2022 (Figure 2).

**Figure 3:**
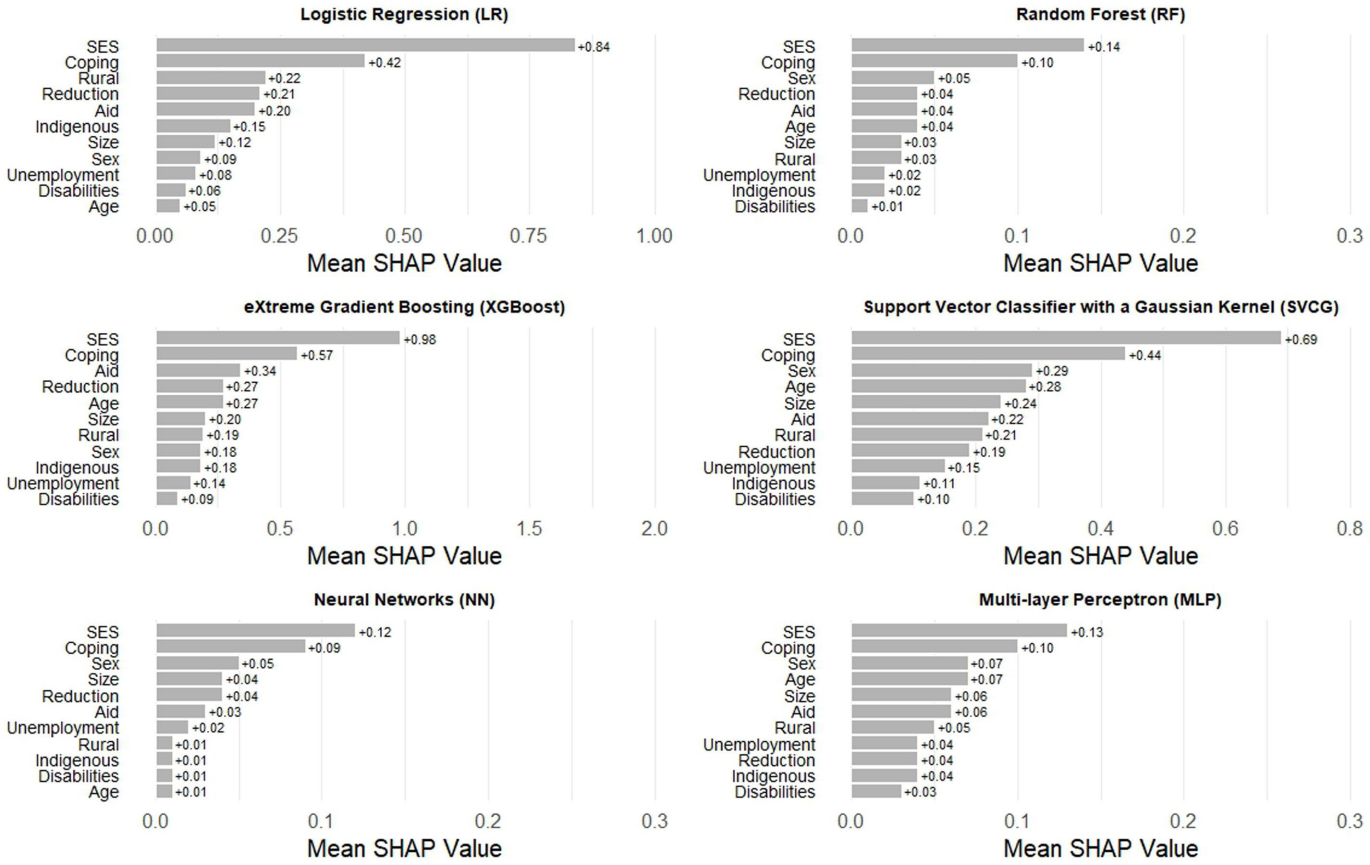
Ranking of SHAP values for the six Machine Learning algorithms in the set of models without baseline household food insecurity.

**Figure 4:**
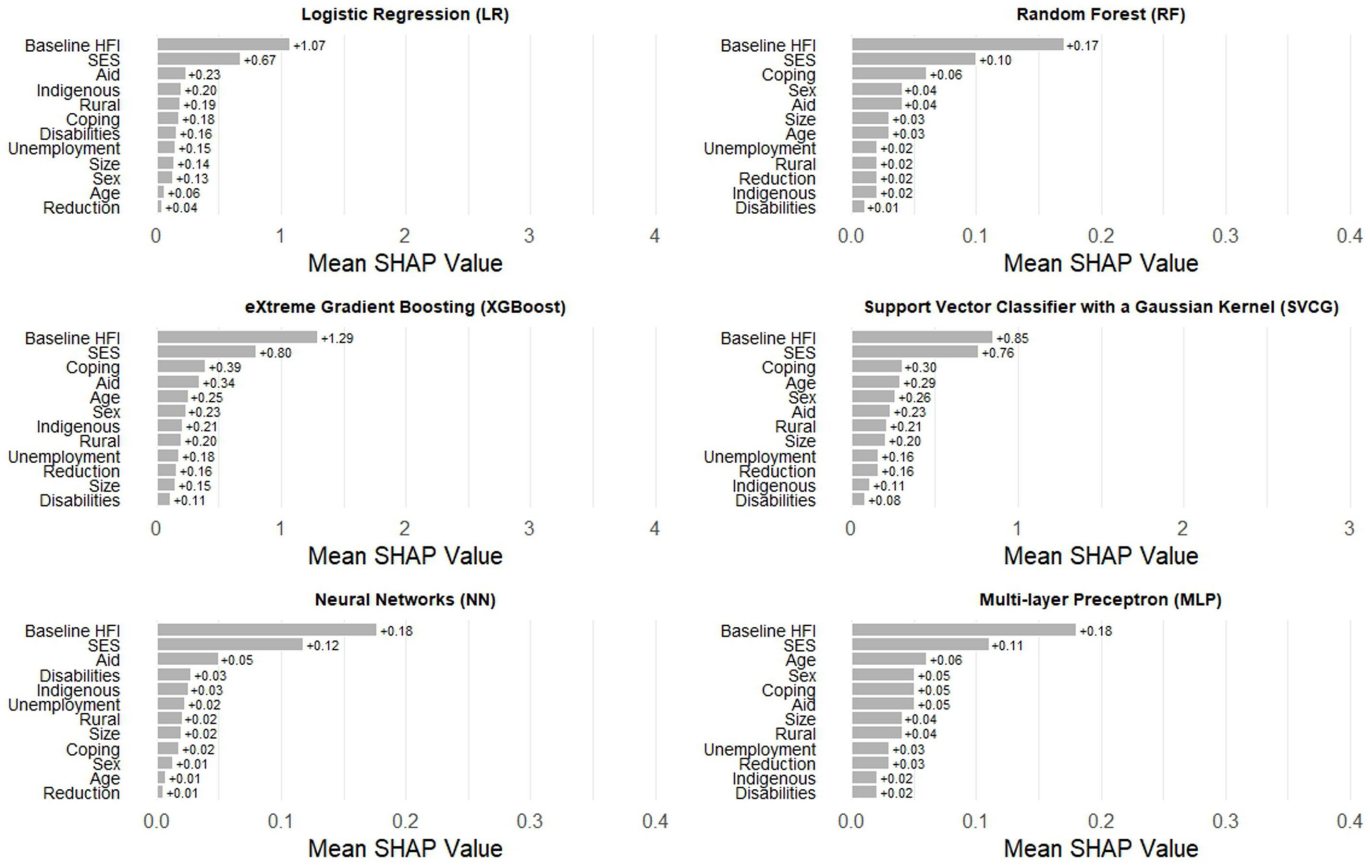
Ranking of SHAP values for the six Machine Learning algorithms in the set of models including baseline household food insecurity.

A strategy to make “black box” algorithms more interpretable is the use of SHAP values, a statistic that shows the relative contribution of each predictor across multiple ML algorithms. In the second set of models, without baseline HFI, the most important predictor in every algorithm is socioeconomic status, a structural variable that is likely invariant since the beginning of the pandemic (Figure 3). The second most consistent predictor is engaging in coping strategies resulting from financial risk and unemployment of a household member during the first month of the pandemic. The rest of the variables shift in importance and consistency across the algorithms. As expected, the most predictive variable in all algorithms in the third set of models was baseline HFI (Figure 4). As with the previous finding, the second most important variable was socioeconomic status. Engaging in coping strategies, as well as receiving aid from the government, were common in most algorithms, but its relevance did not follow a specific pattern.

Analyses with a different specification of the dependent variable—including mild food insecurity–yield different results in the performance metrics (Supplementary Table 2). The prevalence of persistent mild/moderate/severe HFI in 2021 and 2022 is 32.7% – nearly four times than the one without the mild level. The sets of models follow a similar gradient as in the main results, where the lowest performance is observed in the first set of models (*Cohen’s Kappa* is 0.33), the second set slightly improves (*Cohen’s Kappa* is 0.40 on average), and the third model increases in performance (*Cohen’s Kappa* is 0.51 on average). While *sensitivity* and *specificity* are higher than *Cohen’s Kappa,* the relevant conclusion is that—beyond guessing at random (*Cohen’s Kappa* > 0.50)—our independent variables are not suitable to predict a measure of persistent HFI that includes mild food insecurity.

## 4 Discussion

A three-year panel survey allowed us to estimate persistent HFI and to test the predictive power of socioeconomic variables. Our study shows that 8.8% of Mexican households reported having persistent moderate/severe food insecurity in 2021 and 2022. Persistent HFI can be a relevant policy indicator because it identifies households that may be resistant to regular interventions intending to reduce food insecurity. Unfortunately, this is a rarely used indicator that depends on having at least two points in time of longitudinal data (14). The effectiveness of food insecurity interventions needs to be closely monitored because these households might be compounding several deleterious effects related to poverty in a syndemic dynamic that may reduce its impact (41). One example of how to increase these supports is the temporary Child Tax Credit, implemented in the United States of America during the pandemic to help households with minors, and contributed to a reduction by 50% in child poverty (42). Research on persistent HFI—especially during periods without large crises, as the COVID-19 pandemic—would illuminate the design and implementation of adequate interventions targeted at supporting these uniquely challenged households.

The first objective of the study was to compare the predictive performance of multiple ML algorithms. In line with previous research (24), our results show that these algorithms have on average adequate predictive power on persistent moderate/severe HFI, reinforcing the relevance of the ML approach. Random Forest and the Support Vector Classifier with a Gaussian kernel function (SVCG) were the best performing algorithms. Research on HFI could benefit on adopting ML best practices such as partitioning datasets to assess accuracy. Likewise, timely data collection and with sufficient sample size is paramount for the usefulness of these predictions.

A key finding is the role of baseline HFI in the models—which has been identified as the most predictive variable for HFI (21, 28). Focusing on *Cohen’s Kappa,* when baseline HFI is the only predictor, the precision of the algorithms is 0.57. When baseline HFI is absent, the suite of socioeconomic indicators increased precision, on average, up to 0.65. Importantly, the combination of baseline HFI *and* socioeconomic indicators increases the precision to an average of 0.82 (except for Logistic Regression). With both types of variables, these models were able to accurately identify 8 out 10 households reporting persistent HFI. Our model specification confirms baseline HFI is a very relevant predictor and should be collected when possible. In addition, our results show that socioeconomic indicators offer important information beyond baseline estimates of HFI. Predictive models of persistent HFI should aim to have a combination of both types of variables to achieve greater precision. At the same time, our sensitivity analyses show this suite of indicators is not adequate to predict a measure of persistent HFI that includes mild food insecurity. It has been shown that mild food insecurity affected a larger share of the population and during the first months of the pandemic increased at a higher rate than moderate and severe food insecurity, so additional predictors need to reflect a different dynamic (32).

The second objective of the study was to identify the consistently important variables in predicting persistent moderate/severe HFI. There is some consensus over the fact that HFI increased during the COVID-19 pandemic due to a reduction of income that hampered access to food (10). The study assessed income-related predictors to better understand which were more important to identify persistent HFI in a disaster context. The use of six algorithms helped cross-validate the findings and highlight the most prominent predictors, regardless of the analytic assumptions behind single statistical techniques. Besides baseline HFI, two predictors stand out. Socioeconomic status, measured with an assets-based index, was the most consistent predictor, as has been reported in several other studies (13, 18). This is a structural and pre-existent variable that is available in most population surveys and should be included in prediction models. The prominent role of SES reflects that structural poverty is a fundamental determinant of persistent moderate and severe HFI and, if this is a chronic condition, it requires decisive policies to support these households. The second consistent predictor was engaging in coping mechanisms, such as eschewing payments, selling assets, or gaining debt. This indicator is not frequently collected but was important to consider because it relates with the immediate effects of the pandemic on income. Indicators associated with debt should be considered in population surveys as they provide more nuance to the financial situation and the stress in households with HFI. Moreover, these results suggest that short-term financial instruments—like small loans or postponing debt—can be pertinent disaster relief options for future crises. Receiving government aid was a variable that featured in several models, especially when baseline HFI was included, but it was not as consistent as the other two predictors. Contrary to previous research (9), other features of the household were less important for persistent HFI, like the head of household being female or with a disability, age, and household size. Unexpectedly, reductions in income and unemployment were not consistently relevant to predict persistent HFI.

This list of predictors provides important information for future emergency preparedness and response programs, including the relevance of monitoring such variables. In the specific Mexican pandemic context, these findings suggest that government relief actions were insufficient. Mexico’s social policy is mostly based on cash transfers, and, during the pandemic, additional alleviation strategies were nearly inexistent (43). More detailed research could help disentangle the effects of each government program on HFI. Nonetheless, these findings can orient targeting strategies of policy programs aiming to increase food security.

### 4.1 Limitations of the study

The study has some limitations. The definition of “persistent” HFI was limited by ELCSA’s three-month recall period, whereas other measures use a 12-month period, which classify persistence as “often” or “almost every month” (44). This limitation means we are unable to capture fluctuations in HFI status between the two measurement periods. For example, Nord (45) found that, throughout a year, 55% of households experienced one or a few episodes of HFI (some of them lasting several months), 23% experienced low levels of HFI throughout the year with one severe episode, and 22% were persistently food insecure. Likewise, data collected during a 5-year period found that 51% reported HFI once a year, 21% in 2 years, and 14% in 3 years (46). Our scale with a three-month period, measured once a year, is unable to capture this detailed dynamic, which may be relevant in the context of the pandemic, where fluctuations in unemployment and income were common (18). Therefore, we need more research to ensure these patterns hold throughout a year and in the absence of a pandemic.

A larger sample would provide more details on the characteristics of households experiencing persistent HFI. This was partially mitigated using a SMOTE technique, which helps focus the objectives of the algorithms and by adding synthetic cases might artificially increase the accuracy of the algorithms, but unfortunately is unable to provide the needed granularity. Prevalence estimates of persistent HFI might be limited by the normal attrition of panel studies. In this case, the response rate was 51.3 and 60.8%, which is reasonable (31), but may bias prevalence estimates of subsequent survey rounds. Attrition was not random and those who dropped out had a higher moderate/severe HFI (+7%), suggesting our results may be underestimated. These ML algorithms could be even more powerful if secondary data is combined with primary data (24), such as poverty rates, COVID-19 mortality rates, or even food prices (22). However, the present study focused on survey data because there are several high-quality forecasting models available and less longitudinal surveys that may guide variable selection (21). Results would have been stronger if pre-pandemic measurements were collected, if added survey frequency could reflect seasonality, and more variables were considered in the survey, especially for children (47), but the ENCOVID-19 survey began a few weeks after the pandemic started and telephone modality limits survey length. It is desirable to have ongoing panel monitoring systems to have a better understanding of the multiple effects of emergencies and disasters, as well as to inform ongoing policymaking.

## 5 Conclusion

The COVID-19 pandemic had an important impact on household food insecurity (HFI), mostly because income reductions decreased access to food. Throughout the pandemic many households were able to recover, however, the study shows that 8% reported persistent moderate/severe HFI across 2 years. These households are generally characterized by having low socioeconomic status, engaging in coping mechanisms, and receiving government aid. Longitudinal models and powerful predictive algorithms—as the ones in a ML approach—can help improve the identification and monitoring of at-risk households of HFI. Persistent moderate/severe HFI is a relevant indicator that shows the most challenging households for food policy interventions. If we want to reduce the global incidence of HFI we need to account for those who are consistently left behind.

## Data availability statement

The datasets presented in this article are not readily available because the ENCOVID-19 datasets are under a one-year embargo and will be available at ZENODO repository. The datasets analyzed for this study are available upon reasonable request. Requests to access the datasets should be directed to victor.hernandez@ibero.mx.

## Ethics statement

The studies involving humans were approved by Comité de Ética en Investigación de la Universidad Iberoamericana. The studies were conducted in accordance with the local legislation and institutional requirements. The ethics committee/institutional review board waived the requirement of written informed consent for participation from the participants or the participants’ legal guardians/next of kin because the survey was conducted by telephone.

## Author contributions

PG-R: Writing – original draft, Supervision, Project administration, Methodology, Conceptualization. AH-S: Writing – review & editing, Project administration, Methodology, Formal analysis, Data curation, Conceptualization. VL-C: Writing – review & editing, Methodology, Formal analysis. RZ-C: Writing – review & editing, Formal analysis. XG-R: Writing – review & editing, Methodology, Formal analysis. VP-H: Writing – review & editing, Data curation, Conceptualization. MV-C: Writing – review & editing, Conceptualization.
