# Supplementary material for: Predictors of persistent moderate and severe food insecurity in a longitudinal survey in Mexico during the COVID-19 pandemic
Source: Front Public Health. 2024 Jun 26;12:1374815. doi: 10.3389/fpubh.2024.1374815 (PMC11233454; doi:10.3389/fpubh.2024.1374815)
Supplement: Supplementary file 1 [file Table_1.pdf]

Supplementary Table 1. Attrition analysis comparing key variables between the analytic sample and participants lost in follow-up.

Predictor	Categories	Panel participants in 2020, 2021 & 2022 (n = 1,067[1])	Participants who didn´t respond the follow up in 2021 or 2022 (n = 3,613)	*p-value* (95%)
HFI	Food secure	36.78 %	30.11 %	p = 0.000
	Mild insecure	41.70 %	41.26 %	p = 0.825
	Moderate insecure	13.96 %	17.54 %	p = 0.006
	Severe insecure	7.59 %	11.07 %	p = 0.001
Age of interviewee	Age	43.61	40.17	p = 0.000
Sex of interviewee	Woman	53.13 %	57.04 %	p = 0.026
	Man	46.86 %	42.95 %	p = 0.026
Education	1 year	2.43 %	4.04 %	p = 0.018
	3 years	17.90 %	21.20 %	p = 0.021
	4 years	21.55 %	28.84 %	p = 0.000
	5 years	20.52 %	21.89 %	p = 0.361
	6 years	37.58 %	24.02 %	P = 0.000
Size	Number of household members	4.11	4.35	p = 0.000
SES	1	2.90 %	7.11 %	p = 0.000
	2	19.40 %	29.80 %	p = 0.000
	3	14.33 %	16.32 %	p = 0.129
	4	12.65 %	14.55 %	p = 0.128
	5	14.24 %	13.31 %	p = 0.464
	6	18.55 %	12.31 %	p = 0.000
	7	17.90 %	6.55 %	p = 0.000

[1] The sample size in the attrition analysis varies slightly from the sample reported in Table 1. While the sample in Table 1 includes 1,074 participants, the present table reports 1,067 individuals. This is because missing value patterns vary between the two-year models and the attrition analysis.
