# Supplementary material for: Predictors of persistent moderate and severe food insecurity in a longitudinal survey in Mexico during the COVID-19 pandemic
Source: Front Public Health. 2024 Jun 26;12:1374815. doi: 10.3389/fpubh.2024.1374815 (PMC11233454; doi:10.3389/fpubh.2024.1374815)
Supplement: Supplementary file 2 [file Table_2.docx]

**Supplementary material 2. Performance metrics for three sets of models using 2020 data to predict persistent mild/ moderate/severe Household Food Insecurity in 2021 and 2022**

| Set of models | Logistic Regression | Random Forest | XGBoost | SVCG | Neural Networks | MLP |
| --- | --- | --- | --- | --- | --- | --- |
| **Accuracy** |  |  |  |  |  |  |
| 1. HFI | 0.67 | 0.67 | 0.67 | 0.67 | 0.67 | 0.67 |
| 2. SES Predictors | 0.74 | 0.70 | 0.67 | 0.69 | 0.71 | 0.70 |
| 3. SES Predictors and HFI | 0.75 | 0.78 | 0.71 | 0.75 | 0.77 | 0.77 |
| **Cohen´s Kappa** |  |  |  |  |  |  |
| 1. HFI | 0.33 | 0.33 | 0.33 | 0.33 | 0.33 | 0.33 |
| 2. SES Predictors | 0.47 | 0.40 | 0.35 | 0.37 | 0.41 | 0.40 |
| 3. SES Predictors and HFI | 0.50 | 0.56 | 0.42 | 0.51 | 0.54 | 0.53 |
| **Sensitivity** |  |  |  |  |  |  |
| 1. HFI | 0.45 | 0.45 | 0.45 | 0.45 | 0.45 | 0.45 |
| 2. SES Predictors | 0.69 | 0.70 | 0.96 | 0.70 | 0.74 | 0.70 |
| 3. SES Predictors and HFI | 0.69 | 0.81 | 0.91 | 0.80 | 0.73 | 0.76 |
| **Specificity** |  |  |  |  |  |  |
| 1. HFI | 0.88 | 0.88 | 0.88 | 0.88 | 0.88 | 0.88 |
| 2. SES Predictors | 0.78 | 0.70 | 0.40 | 0.67 | 0.67 | 0.69 |
| 3. SES Predictors and HFI | 0.81 | 0.76 | 0.52 | 0.71 | 0.81 | 0.77 |

*Note*: HFI = Household Food Insecurity measured with the adult-version of the ELCSA scale; SES= socioeconomic status measured with the assets-based AMAI index; XGBoost= Extreme Gradient Boosting; SVCG= Support Vector Classifier with a Gaussian kernel function; MLP= Multi-layer perceptron.
